# A conceptual framework for understanding illegal killing of large carnivores

**DOI:** 10.1007/s13280-016-0852-z

**Published:** 2016-11-16

**Authors:** Neil H. Carter, José Vicente López-Bao, Jeremy T. Bruskotter, Meredith Gore, Guillaume Chapron, Arlyne Johnson, Yaffa Epstein, Mahendra Shrestha, Jens Frank, Omar Ohrens, Adrian Treves

**Affiliations:** 1 Human-Environment Systems Research Center, Boise State University, 1910 University Dr., Boise, ID 83725 USA; 2 Research Unit of Biodiversity (UO/CSIC/PA), Oviedo University, Gonzalo Gutiérrez Quirós s/n, 33600 Mieres, Spain; 3 School of Environment & Natural Resources, Ohio State University, 2021 Coffey Rd., Columbus, OH 43210 USA; 4 Office of the Geographer and Global Issues, U.S. Department of State, Washington, DC 20025 USA; 5 Department of Ecology, Grimsö Wildlife Research Station, Swedish University of Agricultural Sciences, 730 91 Riddarhyttan, Sweden; 6 Foundations of Success, 4109 Maryland Avenue, Bethesda, MD 20816 USA; 7 Department of Law, Uppsala University, Box 512, 751 20 Uppsala, Sweden; 8 Smithsonian Conservation Biology Institute, National Zoological Park, MRC 5503, 3001 Connecticut Ave., NW, Washington, DC 20008 USA; 9 Nelson Institute for Environmental Studies, University of Wisconsin–Madison, 30A Science, Madison, WI USA

**Keywords:** Carnivores, Feedbacks, *Gulo gulo*, *Panthera tigris*, Poaching, Social–ecological systems

## Abstract

The growing complexity and global nature of wildlife poaching threaten the survival of many species worldwide and are outpacing conservation efforts. Here, we reviewed proximal and distal factors, both social and ecological, driving illegal killing or poaching of large carnivores at sites where it can potentially occur. Through this review, we developed a conceptual social–ecological system framework that ties together many of the factors influencing large carnivore poaching. Unlike most conservation action models, an important attribute of our framework is the integration of multiple factors related to both human motivations and animal vulnerability into feedbacks. We apply our framework to two case studies, tigers in Laos and wolverines in northern Sweden, to demonstrate its utility in disentangling some of the complex features of carnivore poaching that may have hindered effective responses to the current poaching crisis. Our framework offers a common platform to help guide future research on wildlife poaching feedbacks, which has hitherto been lacking, in order to effectively inform policy making and enforcement.

## Introduction

People and large carnivores are parts of social–ecological systems (SESs), in which human and biophysical subsystems mutually influence one another (Liu et al. 2007a; Carter et al. 2014). In particular, human-caused killing of large carnivores has important consequences on ecosystems and human societies, and exemplifies some important cross-system linkages. Carnivore killing can slow carnivore population growth rates (Liberg et al. 2011; Creel et al. 2015), push some species or populations to the brink of extinction (Woodroffe and Ginsberg 1998; López-Bao et al. 2015; Ripple et al. 2016), and cause unpredictable and wide-spread ecological effects, including the degradation and loss of ecosystem functions and services (Ripple et al. 2014). Carnivore killing can also potentially increase negative human–carnivore interactions (Peebles et al. 2013), aggravate conflict between different groups of people (Krange and Skogen 2011; Duffy et al. 2016), and contribute to debate among the public over carnivore management (Nelson et al. 2016).

Illegal killing (poaching) of carnivores is an especially challenging conservation issue in SESs (Kaczensky et al. 2011). Because in many contexts there are strong incentives to hide, poaching remains poorly quantified (i.e., “cryptic”) and thus its social–ecological causes and consequences are not completely understood (Liberg et al. 2011). A SES framework that reveals key linkages among people, carnivores, and the broader contexts in which they live, and provides guidance on factors needed to evaluate those linkages can therefore be very useful for analyzing poaching of large carnivores. Studies using such a framework in diverse socio-ecological contexts can allow knowledge about poaching dynamics from multiple disciplines to accumulate. For example, Duffy et al. (2016) explored poaching using an integrated approach that considered the complexities of motivations and political–economic contexts so as to make efforts to address poaching more effective, socially, and environmentally just. However, most research on carnivore poaching is still compartmentalized within academic disciplines or takes a reductionist approach to studying relationships (e.g., unidirectional relationships between people and carnivores) and therefore may overlook critical interactions across system components (Milner-Gulland 2012; Larrosa et al. 2016). Furthermore, although a number of SES frameworks have already been developed to analyze and address various environmental problems (e.g., Ostrom 2009; Scholz et al. 2011), none have been developed to address wildlife poaching, and the existing frameworks are not adequately designed to do so. Existing frameworks typically consider how social and ecological processes, mediated through institutions, influence and are influenced by the production of a natural resource.

We have two objectives in this paper. First, we review the literature to develop a conceptual SES framework for understanding the multiple factors and feedbacks influencing carnivore poaching. Second, we apply the conceptual SES framework to two case studies: (1) wolverines (*Gulo gulo*) in northern Sweden and (2) tigers (*Panthera tigris*) in Laos. The application of our SES framework to the two cases underscores how conservation interventions are more likely to be effective when both proximal and distal social–ecological causes and consequences of human–wildlife interactions are accounted for—a process that takes time and meaningful engagement with various stakeholders.

### Conceptualizing wildlife poaching

“Poaching” is a contested term and may have different meanings in different contexts. For example, the National Tiger Action Plan for Lao PDR 2010–2020 uses the term to describe the direct illegal killing of tigers for commercial trade, as opposed to other motivations to kill tigers such as risk perceptions, beliefs about tigers and the people that kill tigers, perceived personal rewards, or the severity and locations of tiger incidents (Inskip et al. 2014). Most international wildlife conservation agreements and many national species protection laws do not use the word poaching; instead, they simply describe what actions are prohibited. However, one common usage of the term is the illegal killing or taking of wildlife, and for the sake of convenience and consistency that is the definition we adopt here (Musgrave et al. 1993).

Many of the world’s anti-poaching laws are enacted pursuant to international agreements inspired by the Stockholm Declaration (Sohn 1973) after the United Nations Conference on the Human Environment held in Stockholm, Sweden, in 1972, which called upon governments and their populations to take action to protect the natural resources of the earth, including the air, water, land, flora, and fauna, for the benefit of the present and future generations. Important treaties following in the wake of this declaration include the Convention on International Trade in Endangered Species of Wild Fauna and Flora (CITES) of 1973, to which currently 175 countries have acceded, which requires its parties to take measures to penalize the trade in and or possession of protected species, and the Bonn Convention on the Conservation of Migratory Species of Wild Animals of 1979, which requires its 120 parties to prohibit the taking of endangered migratory species. The 96 parties to the 1992 Convention on Biological Diversity agreed to take measures to conserve species as components of biological diversity. In 2015, 33 parties agreed to the Kasane Statement, which articulated various actions that the parties should take to eradicate both the demand for and supply of illegal wildlife products. These international agreements have spurred protective measures in many jurisdictions on the one hand, but on the other have few enforcement mechanisms to ensure that conservation actually takes place.

Treaties and national laws are an important tool for protecting species. However, the mere enactment of laws is often insufficient to impact poaching, particularly in developing countries where laws may be unknown, resources for detecting violations are insufficient (Rowcliffe et al. 2004), or corruption is pervasive (Gore et al. 2013). Conservation laws can have the opposite impact than intended if they are seen as illegitimate or undemocratic (Essen and Allen 2015). In some cases, regulations and other interventions intended to reduce risks from poaching are considered unjust and carry with them their own set of risks to local peoples (Duffy et al. 2016). Certainly, the legitimacy and democracy of anti-poaching rules are questionable if they do not serve the public interest (Smith 2012). We acknowledge that what distinguishes poaching from legal hunting might be influenced more by socio-political, human geography, or historical factors, including power asymmetry, histories of conflict between groups over land tenure, and human insecurity, than by its environmental consequences (Bekoff 2013). However, rather than question the legitimacy or democracy of anti-poaching rules on a case-by-case basis, we assume throughout this paper that poaching, as defined by some legal authority, poses some measurable risk to effective wildlife conservation and that conservation includes both humans and wildlife. While this assumption may obfuscate cases in which anti-poaching rules might be perceived as unjust, in general, we contend that this assumption holds true for a globally diverse suite of poaching cases. The conceptual framework for analyzing poaching, described in this paper, is therefore not intended to reflect on the appropriateness or fairness of anti-poaching rules, which is beyond the scope of this work, but rather to guide research on ways to reduce risks emerging from poaching and its deleterious effects on wildlife populations and ecosystems across contexts. A small but growing body of scholarship is beginning to attend to these issues, and the interested reader is directed to Duffy (2016) and Phelps et al. (2016). These studies, and others, demonstrate key critiques of conservation rules in the face of poaching, namely that strict anti-poaching measures may violate human rights, disproportionately blaming local communities for negative impacts of poaching. On the other hand, others caution that these arguments sometimes exclude nonhuman, or prioritize human, rights in a way that depoliticizes the need for legal protection of wildlife (Beirne 1999; Wyatt 2009).

### Characteristics of our conceptual framework

**Fig. 1:**
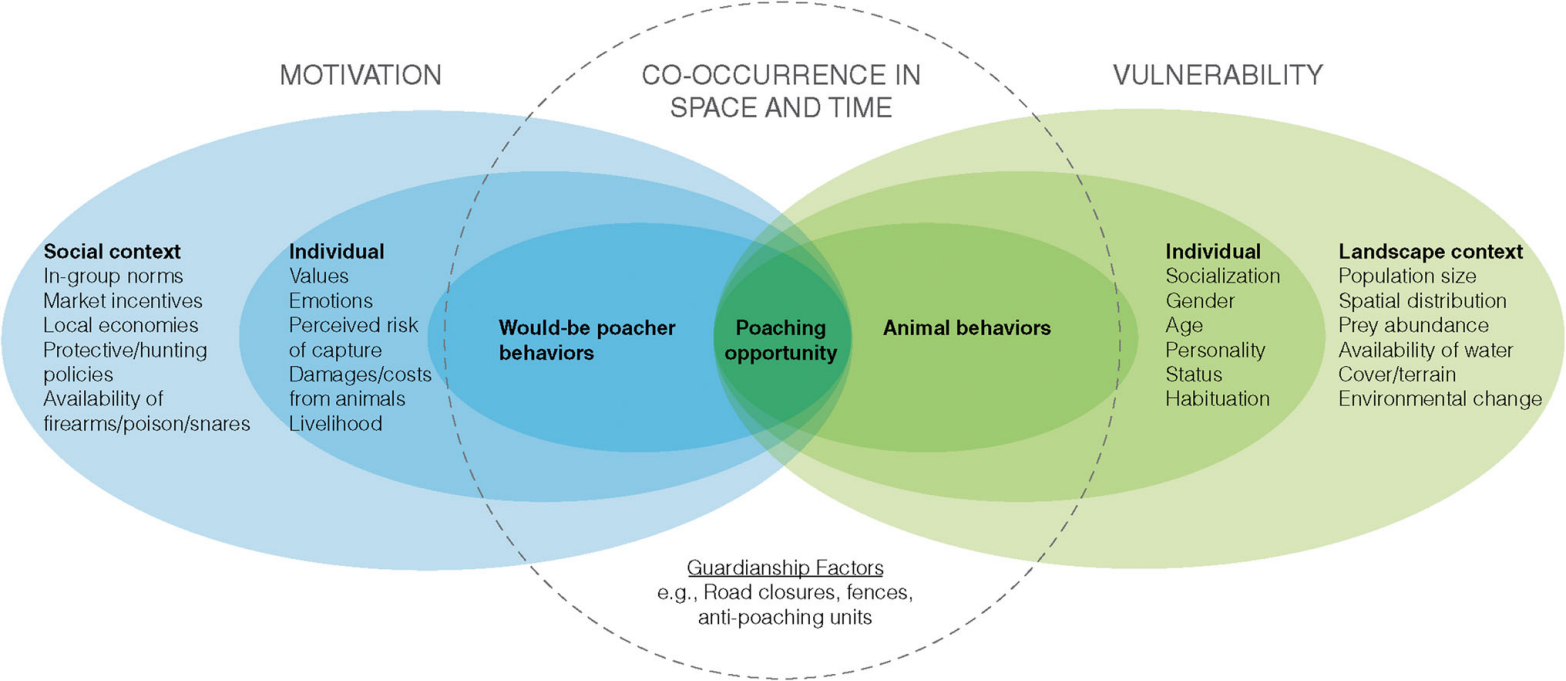
Social–ecological system framework for carnivore poaching. Human–carnivore interactions span different levels, indicated by *overlapping circles*. The area within the *dashed circle* indicates the co-occurrence of would-be poachers (or their tools such as traps) and vulnerable animals in space and time. Co-occurrence is influenced by guardianship factors, such as road closures, fences, and anti-poaching units, among many other related factors. Factors listed in each level are frequently shown to influence poaching dynamics; however, other factors not listed here might also relate to illegal killing of carnivores or other wildlife species

In our framework, poaching of large carnivores is viewed as occurring within a nested, multi-level SES (Fig. 1). The factors in the inner-most levels are most proximal to the physical act of illegal killing and reflect the characteristics (e.g., lack of patrols) immediately affecting the opportunity for poaching to arise at a given place and time. The factors in these levels are based largely on studies in situational crime prevention, which is an approach designed to provide practical guidance in reducing criminal opportunities (Clarke 1995, 2008), such as informing where the location and frequency of patrols by law enforcement officers may be most effective. Factors in the intermediate levels reflect the individual characteristics that may directly motivate a person to poach or increase an animal’s vulnerability to poaching (Fig. 1). The factors in these levels are based largely on studies in human psychology and animal behavior (Kahler and Gore 2012; Kahler et al. 2013; Kertson et al. 2013; Steyaert and Kindberg 2013), and represent points in the system where a suite of conservation interventions can (and have) focus, including education programs to inform people about hunting regulations. Finally, the outer-most levels include more distal factors influencing poaching and reflect aspects of the broader social and ecological contexts in which human societies and wildlife populations interact (Fig. 1). The factors in these levels are based largely on studies in social norms and political science as well as wildlife population and community ecology (Forsyth and Marckese 1993; Ripple and Beschta 2006; Lute et al. 2016).

Having the different levels of our framework interlinked underscores the likelihood of feedbacks in systems of human–wildlife interactions that potentially influence poaching opportunities. Recent work has highlighted the importance of addressing feedbacks in complex coupled systems (Hull et al. 2015) and begun categorizing the causal mechanisms by which conservation interventions give rise to unintended feedbacks (Larrosa et al. 2016). Although empirical evidence of feedbacks affecting or affected by poaching is sparse, likely due to a lack of studies integrating social and ecological dynamics, there are examples of feedbacks that directly affect wildlife population viability. For example, Nijman et al. (2009) showed how highlighting the conservation plight of the Javan hawk eagle (*Spizaetus bartelsi*) inadvertently increased the demand for and trade of this bird of prey (Nijman et al. 2009). Similarly, Wilkie and Godoy (2001) show how enforcement of anti-poaching laws increased prices of wildlife and, contrary to its desired effect, induced more people to enter the market and increase killing levels (Wilkie and Godoy 2001).

SESs are shaped by a complex constellation of factors that change dynamically and sometimes in surprising ways (Liu et al. 2007b). For example, changing economic circumstances in certain countries can increase demand for wildlife parts from other countries (Duffy et al. 2015). Or, the practices and policies regulating how a certain group of people interact with wildlife can change abruptly after having remained unchanged for a long period of time (West and Brockington 2006). Instead of trying to capture all possible root drivers of illegal killing, our framework focuses on those factors that might enable conservation professionals to analyze and reduce poaching at spatiotemporal scales most relevant to them.

### Comparing our framework to other SES frameworks

Our framework is both analysis- and action-oriented. Like some other SES frameworks, such as the Ecosystem Services framework (Costanza et al. 1997), our framework is primarily meant to help organize relevant factors identified in theories and empirical research by biophysical and social scientists. The framework therefore provides a structure for synthesizing data for improving our understanding of poaching in a coupled system. The framework is also action-oriented, because it allows the placement of anti-poaching policies within a nested SES, thereby facilitating the assessment of policy efficacy at multiple levels. By providing information to improve a particular situation (i.e., reduce risks associated with poaching), our framework is akin to other frameworks, like the Sustainable Livelihood Approach or the Driver, Pressure, State, Impact, Response framework (Scoones 1998; Carr et al. 2007).

Although carnivore poaching has consequences on both the social and ecological systems, we have organized the framework to guide research on ways that could potentially reduce the negative effects of poaching on wildlife and ecosystems. Our framework is therefore distinct from many other SES frameworks, such as the Human Environment Systems Framework or Vulnerability framework, which tend to have an anthropocentric perspective (Turner et al. 2003; Scholz et al. 2011; Shoreman-Ouimet and Kopnina 2016). That is, they consider the ecological system primarily as a provider of services that increase human well-being, and their goal is facilitating the sustainable provision of those services for people (Ostrom 2009; Scholz et al. 2011). In contrast, our SES framework focuses on understanding the “production” of an illegal human behavior (i.e., poaching) affecting organisms (i.e., large carnivores) that provide both ecosystem services (e.g., ecotourism revenue, regulating trophic interactions, and cultural values) and disservices (e.g., threats to livestock, competition for game, and human safety). That benefits and risks associated with large carnivores are heterogeneously distributed among segments of human societies makes carnivore conservation, and especially reduction of poaching, challenging.

### Poaching opportunity and proximal causes of poaching

The proximate causes of carnivore poaching arise from physical interactions between “*would*-*be poachers*” and vulnerable animals sharing the space (Fig. 1). Routine Activity Theory (Eliason 2012) posits that poaching opportunity is a function of three interacting components: motivated would-be poachers, suitable targets (i.e., vulnerable animals), and lack of guardianship (e.g., lack of strong law enforcement and community-based anti-poaching units). For example, animals that are elusive, spatially dispersed, less abundant, or otherwise difficult to locate, such as large carnivores, may require substantial effort by poachers to encounter. Accordingly, poachers utilize certain tools (e.g., baits, traps, pitfalls, nets, and poisons) to capture/kill animals even when poachers and their targets are not in the same place at the same time. Human accessibility and activities in areas with wildlife can increase the ease with which would-be poachers can find and dispatch animals (Kerley et al. 2002). On the other hand, anti-poaching forces can restrict spatial and temporal access of poachers to wildlife, for example, through barrier fencing or closing logging roads (Laurance et al. 2009). Furthermore, anti-poaching forces may increase the likelihood of apprehension of poachers through improved patrolling strategies (e.g., use of un-manned aerial vehicles or surveillance cameras) and intelligence derived from local informants (Linkie et al. 2006; Steinmetz and Srirattanaporn 2014).

Several poaching mitigation strategies are being developed or have been employed, which incorporate knowledge of feedbacks among these proximal factors. For example, models based on game theory can assist conservation practitioners in devising more effective patrol strategies (e.g., spatial locations and frequencies) by simulating the dynamic interactions and adaptations between patrols and poachers while considering the movement patterns of the wildlife species of interest (Yang et al. 2014). However, poaching opportunity and its proximate causes are underlain by a complex array of motivations to poach and factors affecting animal vulnerability to poaching that cannot necessarily be pinned down to a specific time and place (Challender and MacMillan 2014). Interventions focusing only on the proximate causes, although necessary, can therefore only partially address the poaching crisis (Margoluis et al. 2013). Below we describe individual-level factors, such as human motivations and animal life-history traits also influencing poaching opportunity.

### Individual-level factors influencing poaching opportunity

There are a number of factors that might motivate an individual to become a poacher (e.g., costs and benefits, values, emotions, livelihoods; Fig. 1). For example, economic costs incurred from large carnivores, particularly due to livestock loss, have been commonly referenced as an important driver for illegal killing (Treves and Karanth 2003; Johnson et al. 2006; Zabel and Holm-Müller 2008). In addition to economic costs, emotional responses from local people, such as fear (Flykt et al. 2013), have been shown to drive carnivore poaching (Nie 2003; Salvatori and Linnell 2005). In contrast, the high prices that wild tiger parts fetch on national and international black markets (Gratwicke et al. 2008) encourage commercial poachers (many of which are increasingly nonlocal) across the tiger’s remaining 13 range countries (Dinerstein et al. 2007).

Many theories exist to explain noncompliant behavior, like poaching (Keane et al. 2008; St. John et al. 2010; Klöckner 2013). The Reasoned Action Approach is a popular model for predicting, explaining, and changing human behavior and can integrate many of the main factors underlying intentions to poach carnivores. This model suggests that behavioral intentions are influenced by behavioral, normative, and control beliefs (Fishbein and Ajzen 2010; Arias 2015). In the case of carnivore poaching, behavioral beliefs are associated with the costs and benefits of noncompliance with anti-poaching rules. Studies on behavioral beliefs thus often use economic models of compliance with anti-poaching laws. In such models, benefits from poaching are typically valuated monetarily (e.g., reduction of livestock loss, income from selling body parts) and the costs related to the probability of being detected and punished and the severity of the punishment (Keane et al. 2008, 2012).

Normative beliefs are also important for determining compliance (St. John et al. 2010; Arias 2015). Normative beliefs can be categorized into two basic types: personal and social. Personal norms describe moral beliefs about what is right and wrong to do and can be related to social norms (Klöckner 2013), which are the collective norms that guide individual behavior (see discussion on social norms in section “Broader social and landscape factors influencing poaching opportunity”). Although personal norms are not part of the Reasoned Action Model, they may indicate whether someone feels morally obligated to comply or regret noncompliance or regret the act of killing an animal. Personal norms have been shown to help explain pro-environmental and prosocial behavior (Klöckner 2013). However, to our knowledge no studies have evaluated the effects of personal norms on carnivore poaching behaviors, although Browne-Nunez et al. (2015) found that in many cases people in Wisconsin participating in social surveys expressed attitudes, beliefs, and behavioral intentions that could be categorized as pro-poaching.

Control beliefs are people’s perceptions of what limits or facilitates a particular behavior (St. John et al. 2010; Arias 2015). Knowledge, skills, time, money, and equipment are examples of factors influencing perceived control. Information about the illegality of killing certain wildlife species, for instance, can remove knowledge barriers from hunters unaware of rules (Arias 2015). Furthermore, banning the possession or importation of gear that facilitates the illegal killing of wildlife (e.g., high-powered rifles) can obstruct access to that equipment by would-be poachers. However, the effects of such interventions have to be monitored closely, as unintended feedbacks may result, such as the increasing use of other illegal methods for killing wildlife that are cheaper and simpler (e.g., wire snares, poison).

As with human motivations to poach being influenced by individual-level factors, various individual-level traits of carnivores, such as age, sex, or status, influence their vulnerability to be poached (Fig. 1). For example, different animal personalities will affect poaching risk, with bold individuals probably being more vulnerable to poaching compared to shy individuals because they take more risks (Bremner-Harrison 2004; Sih and Del Giudice 2012). Food-conditioned or human-habituated individuals may also be more vulnerable to poaching (Whittaker and Knight 1998). Furthermore, large carnivores living in social units or those that aggregate at common use areas may be easier to poach than other species. For example, wolves (*Canis lupus*) repeatedly using den and rendezvous sites and vocalizing in the vicinity are more vulnerable to potential poachers, especially those actively searching for litters or aiming to eradicate entire wolf packs (Wydeven et al. 2004; Fernández and Azua 2010). On the other hand, since large carnivore males are often more conspicuous (e.g., vocal behavior) and usually have larger home ranges and longer dispersal distances than do females (Waser and Jones 1983; Mikael 1989), males may be more vulnerable to poaching than females.

Importantly, from an evolutionary point of view, large-bodied animals are rarely adapted to high adult mortality rates (Darimont et al. 2015; Krofel et al. 2015). Poaching of large carnivores can therefore have significant behavioral consequences, such as destabilization of social organization, sexually selected infanticide, and changes in habitat selection patterns (Wielgus and Bunnell 1994; Wielgus et al. 2001; Borg and Brainerd 2015), as well as population consequences, such as reduction of emigration/immigration and the creation of source-sink population dynamics (Woodroffe and Ginsberg 1998; Festa-Bianchet 2003; Woodroffe and Frank 2005; Adams and Stephenson 2008). Understanding behavioral and population consequences sheds light on general patterns of human–carnivore interactions. For example, lethal management of cougars (*Puma concolor*) is a common response to livestock predation. However, a previous study suggests that livestock losses can increase following lethal harvests, as the cougar’ social organizations are disrupted (Peebles et al. 2013).

Individual-based factors of both people and wildlife can interact and feed back to influence poaching. For example, although spotted hyenas (*Crocuta crocuta*) and leopards (*Panthera pardus*) kill livestock belonging to Maasai people in Kenya in high numbers, lions (*Panthera leo*) appear to be much more vulnerable to illegal killing (Kissui 2008). In part, this may be because lions tend to kill cattle, which evokes a stronger emotional response from the Maasai than the loss of smaller livestock. Also, lions are more likely to defend their kill than other predators, which increases the probability of a Maasai-lion encounter and creates a feedback that facilitates confrontations with Maasai and reinforces the cultural importance of displaying bravery by spearing lions (Kissui 2008). Thus, economic tools to reduce the costs (e.g., livestock depredation) of living near lions, such as compensation schemes, insurance, and revenue-sharing programs, may be less effective than expected because of factors (e.g., culture) that shape individual motivations to kill carnivores (Hazzah et al. 2014). In the following section, we highlight several ways in which the broader social and landscape contexts influence poaching and create important feedbacks.

### Broader social and landscape factors influencing poaching opportunity

Factors associated with human social context, such as in-group (i.e., a group of people that one strongly identifies with) norms about poaching, cultural uses of wildlife, and natural resource-protective policies, shape individual motivations to poach (Fig. 1). A normative perspective focuses on the degree to which someone views a conservation policy as appropriate, legitimate, and consistent with the behaviors and expectations of others as well as how the actor will be viewed by others if (s)he poaches (Kinzig et al. 2013; von Essen et al. 2014a). Studies have found that rules in use (i.e., “rules in action” as opposed to “law on the book”), comprising norms of acceptable behavior reinforced by social pressure, govern the timing and method for harvesting wild species independent of the rule of law (Gore et al. 2013). For example, despite laws prohibiting it, lion killing by young Maasai men in Kenya occurs as part of a cultural tradition; killing has declined due largely to normative interventions that have sought to stigmatize lion killing (Hazzah et al. 2014). Poaching can sometimes be viewed as a “folk” crime or unimportant crime, characterized as socially acceptable but politically incorrect to investigate too closely (Muth and Bowe 1998). Broad socio-political conditions can also trigger and perpetuate poaching. For example, poaching of wolves in Sweden is sometimes rationalized by viewing the wolf as an immigrant or government-bred hybrid rather than of “pure” Swedish stock (von Essen et al. 2014b). Furthermore, poaching has emerged in the past as social defiance against local natural resource management practices that were considered illegitimate (Nie 2003; Bruskotter et al. 2011; Becker et al. 2013; von Essen et al. 2014a).

Likewise, factors related to the broader landscape context influence the vulnerability of animals to poaching (Fig. 1). For example, adult male brown bears (*Ursus arctos*) used remote areas, whereas adult females and some sub-adults used areas closer to roads or human-dominated areas (Steyaert and Kindberg 2013). In northern regions, snow cover can facilitate poaching as it increases the detectability of carnivores and their tracks. For example, adult wolverine survival in northern Scandinavia was lower during the snow season, presumably because people could track and poach them easily from snow machines (Persson et al. 2009). In more tropical regions, the onset of the dry season can increase carnivore vulnerability to poaching. During the hot-dry season, animals will more predictably aggregate near diminishing water sources and they become more easily detected due to reduced vegetation cover (Becker et al. 2013). The number of snares detected in the field by anti-poaching patrols in Zambia’s Luangwa valley, for instance, was highest in the hot-dry season, with significant impacts on mortality rates of lions and African wild dogs (*Lycaon pictus*) (Becker et al. 2013). Furthermore, changing landscape factors can alter human–carnivore interactions, with implications for carnivore poaching. For example, in the past, livestock depredation by lions in Kenya commonly occurred during the wet season when lions moved from national parks into communal lands to follow migrations of their wild prey (Kissui 2008). Most recently, however, the spatial distribution of the lion population in Kenya has changed, with more and more lions living outside parks and preying on livestock year round (Hazzah, L., personal communication). Greater likelihood of livestock depredation in human-occupied areas outside parks may aggravate poaching of lions aimed at reducing the threat.

When individual- and broader-level factors driving poaching are considered together, it becomes more apparent that poaching is influenced by interactions across levels as well as within levels. For example, the likelihood that a specific method (e.g., firearm, wire snare) is used to kill carnivores depends not only on policies controlling the availability of each method to would-be poachers but also on carnivore behavior, abundance, and habitat preferences. Although firearms are a powerful weapon, it is very difficult to track and shoot an elusive tiger in dense tropical forest habitat versus setting and baiting snares, which are effective and difficult to detect by anti-poaching units. Poaching interventions that account for factors at multiple levels are therefore needed. For example, although poaching of tigers for the global market is a persistent challenge to conservation, multiple factors operating at different levels (e.g., integration of knowledge about tiger and forest ecology, social norms and institutions, as well as improved enforcement strategies) have significantly enhanced poaching interdiction and deterrence in Nepal (Nowell 2012). Below we present more specific examples of feedbacks related to carnivore poaching and illustrate how factors associated with all levels of the framework play a role in shaping poaching dynamics.

## Feedbacks and effects of policy interventions

**Fig. 2:**
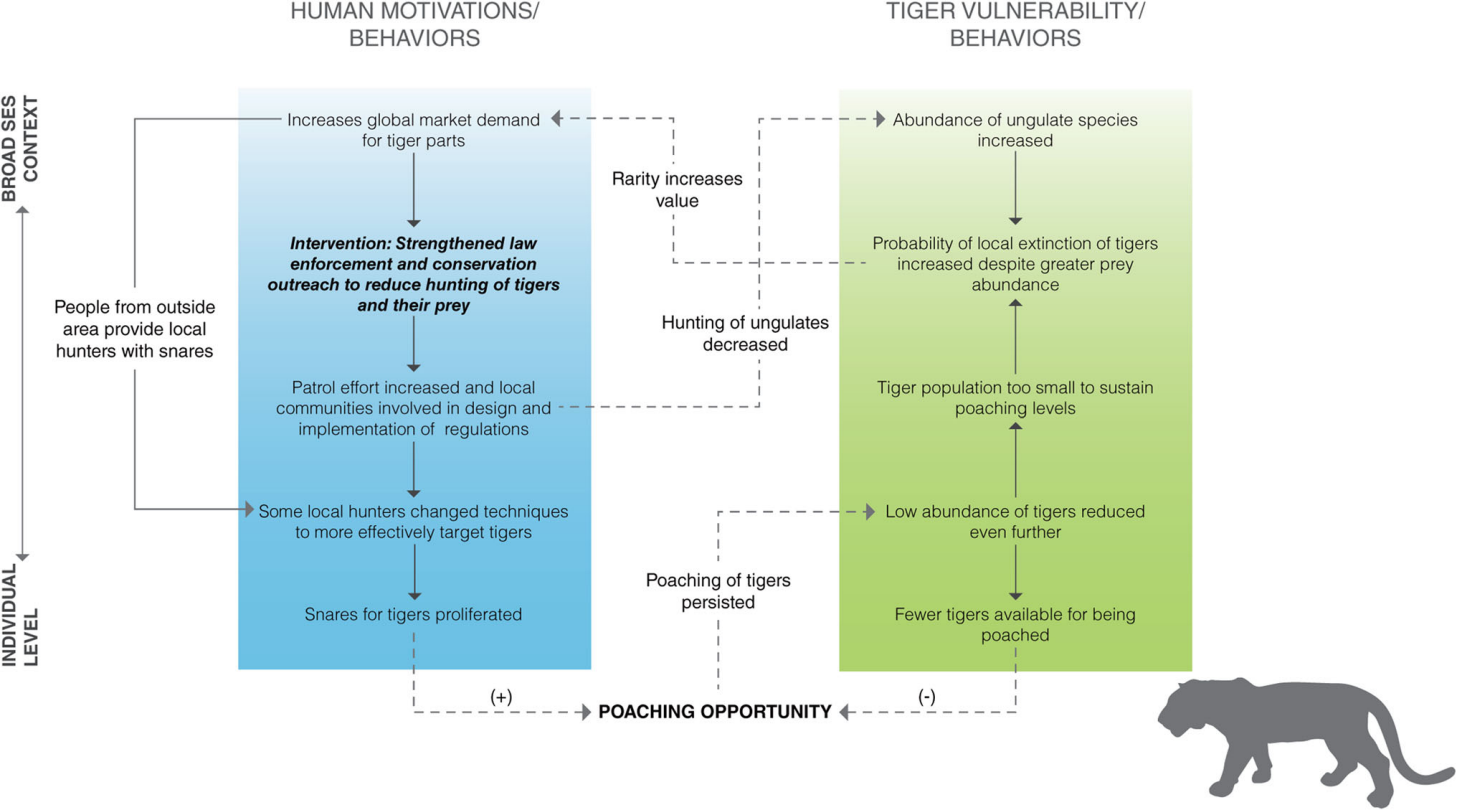
Social–ecological system diagram illustrating how various social and ecological factors, their interactions, and feedbacks compromised the effectiveness of an intervention designed to reduce tiger poaching in Nam Et-Phou Louey National Protected Area, Laos. *Dashed lines* indicate key interactions across the social and ecological subsystems that form feedbacks

**Fig. 3:**
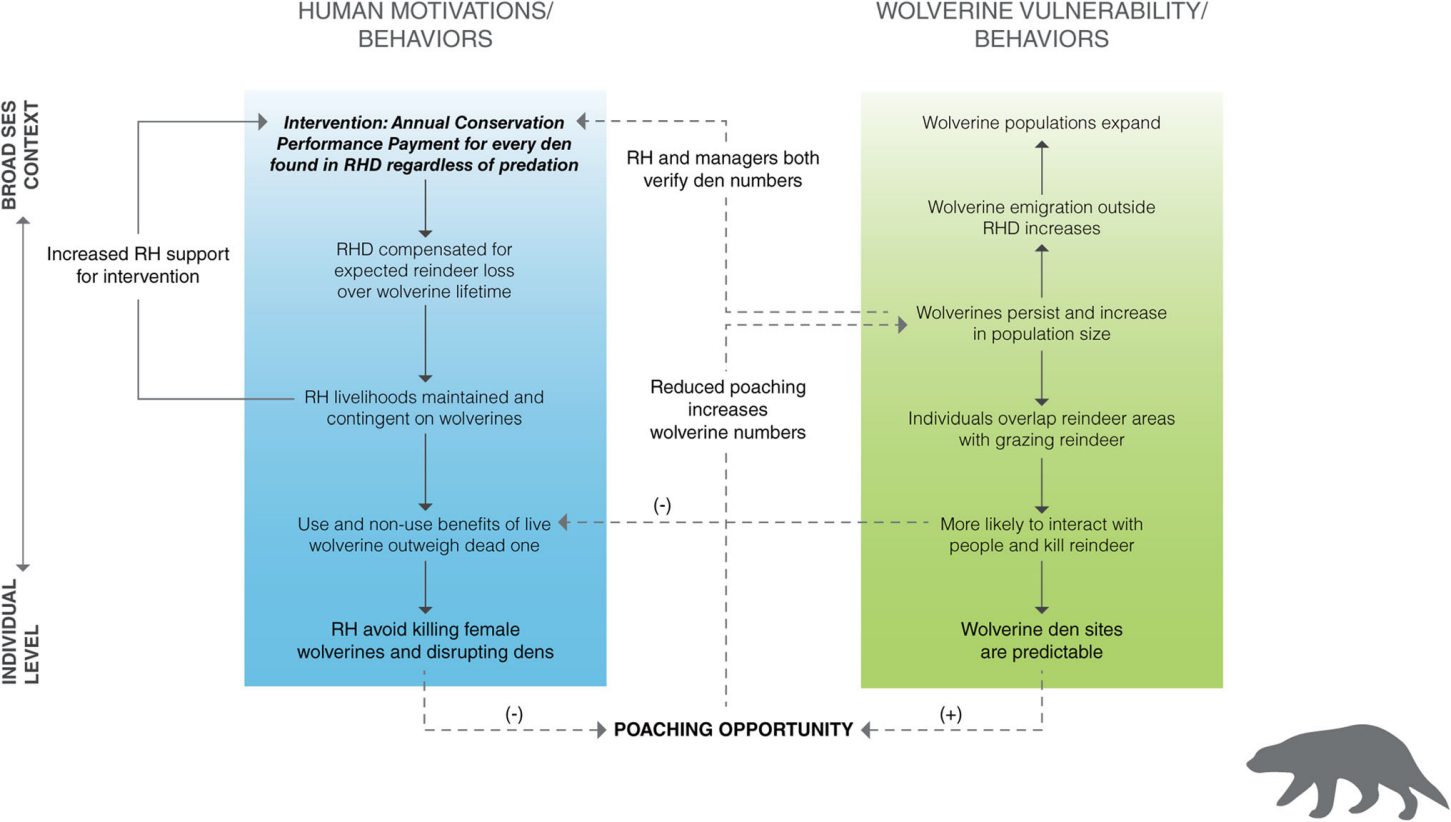
Social–ecological system diagram illustrating how various social and ecological factors, their interactions, and feedbacks enhanced the effectiveness of an intervention designed to protect human livelihoods and increase wolverine numbers in northern Sweden. *Dashed lines* indicate key interactions across the social and ecological subsystems that form feedbacks. *RH* reindeer herders, *RHD* reindeer herding district

A better understanding of the feedbacks within and across levels can improve the effectiveness of policy interventions aimed to reduce poaching. The information used to construct each SES diagram (Figs. 2, 3) for the examples described below is based mostly on empirical data as well as hypothesized links derived from the scientific literature. Despite very different social–ecological contexts and spatial scales, these two examples demonstrate how the SES framework for carnivore poaching can be used to pinpoint how and why anti-poaching interventions are more or less effective.

### Tigers in Nam Et-Phou Louey National Protected Area, Laos

Established in 1993, the Nam Et-Phou Louey National Protected Area (NEPL NPA) is an IUCN Category VI protected area where a proportion of the area is open to sustainable use of natural resources (Berkmuller et al. 1995; Johnson 2012). There is a long history of human settlement with most villages engaged in subsistence activities and limited integration into the market economy. Rice is the staple food and is primarily produced through rotations of shifting cultivation on steep mountainous slopes. Livestock, a principle source of cash income, graze freely in forested areas and grasslands, sometimes hours away from the villages. Wild foods still play an important role in household food consumption, with most of the meat and vegetables coming from the wild, relative to foods purchased or domestically produced (ICEM 2003; Johnson et al. 2010). Baseline camera trap surveys from 2003–2004 indicated that relative abundance of large ungulates was low throughout the NPA, while small prey was significantly higher where human density was lower. The estimated tiger density was 0.7/100 km^2^ with significantly lower abundance where human population and disturbance were greater (Johnson et al. 2006).

Three interventions were implemented in 2005 to reduce poaching on tigers and their prey (wild ungulates) in NEPL NPA (Johnson et al. 2016). The interventions consisted of (i) strengthening law enforcement activities by increasing foot patrol frequency and coverage and (ii) working with local government, communities, and the military, to establish and enforce inviolate core zones where tigers and prey would not be poached (Fig. 2). Furthermore, (iii) a Wildlife Crime Unit was created to facilitate public reporting and apprehension of wildlife crime (Johnson et al. 2016). During 2005–2009, increasing foot patrol effort led to a significant decrease in the detection of poaching tools (e.g., snares) per 100 km patrolled. However, during 2009–2012, there was an exponential proliferation in confiscation of wire snares (Johnson et al. 2016). Relative to the baseline survey in 2003/2004, in 2012 the relative abundance of all ungulate species significantly increased, ranging from over a twofold increase (2.77–6.97) for muntjac (*Muntiacus muntjak*) to over a fourfold increase (0.25–1.2) for Sambar deer (*Cervus unicolor*). Yet, despite greater prey abundance, there was a sevenfold decline (0.24–0.03) in mean relative abundance index for tigers over the same period (Johnson et al. 2016).

Without being able to separate the impact of each intervention, the mixed outcomes from these interventions (increase in wild prey, but a decrease in tiger abundance) underscore how cross-scale linkages (e.g., local to global) in the SES likely led to different underlying human motivations to poach ungulates versus tigers, which in turn led to divergent trends in their populations. Locals targeted ungulates mainly for subsistence and local trade. Engaging local communities and clarifying regulations on when and where people could legally hunt less-threatened ungulates for subsistence (i.e., outside the inviolate core zones) likely bolstered public support for the protection of ungulate source populations within NEPL NPA, while the increased enforcement was sufficient to reduce pressure on ungulates within the core zones for the less lucrative local trade. In contrast, global demand for tiger products (e.g., in traditional medicines) created strong economic incentive for locals to poach tigers and sell their parts to traders. This was facilitated by traders from Vietnam and China altering the control beliefs of a subset of local hunters by providing them with snares for tigers (Fig. 2), which were uncommon in the area in the past (Johnson et al. 2016). Furthermore, the behavioral beliefs about poaching tigers among would-be poachers were likely influenced by the relatively low likelihood of being prosecuted and convicted of tiger poaching (Johnson et al. 2016). Instead of reducing poaching of tigers, as had happened with ungulates, some local hunters therefore adapted to the modified foot patrol strategies by more effectively targeting tigers and thus reducing tiger abundance within NEPL NPA. Fewer tigers likely make it harder for poachers to find and kill them; however, poaching rates were unsustainable given the already small tiger population in the core zone (Johnson et al. 2006). Consequently, the probability of tigers being extirpated from NEPL NPA has increased despite the implementation of law enforcement interventions and an apparent increase in wild prey. As demand for large carnivores with high economic value is tied to their population size, we would expect a feedback in which the demand for tigers increases with their rarity (Courchamp et al. 2006), causing poaching effort to increase and further reduce tiger population size (Chen 2015).

### Wolverines in northern Sweden

Reindeer herding is central to the livelihoods of the Sami people in Sweden (Zabel and Holm-Müller 2008). The first written evidence of reindeer husbandry in Scandinavia is from 800 A.D. (Lundmark 1982), but exactly how far back the tradition goes is unknown. The number of reindeer herders in Sweden is rather stable at 4500, owning a total of 240 000 reindeer (Statistics Sweden 2016). For about a century, nomadic extensive reindeer husbandry has been far more common than intensive reindeer husbandry (Manker 1944). Extensive reindeer husbandry is advantageous as it demands less man-hours and also results in healthier reindeer as the animals graze over larger areas.

In the past, reindeer herders killed large carnivores, including wolverines, in response to real or perceived reindeer losses from depredation (Persson et al. 2009). Ex post compensation schemes (payments made after livestock loss) have been used to reduce carnivore-related costs to herders (Nyhus et al. 2005). From a purely economic self-interest or instrumental perspective, compensation for livestock losses should eliminate much of the motivation to kill large carnivores and therefore reduce poaching. However, such schemes face numerous criticisms, such as problems of trust and legitimacy, and sometimes produce unintended outcomes (Nyhus et al. 2005; Zabel and Holm-Müller 2008). For example, people use less optimal livestock husbandry practices because compensation is not contingent on livestock protection measures, a situation economists refer to as a “moral hazard” (Nyhus et al. 2005).

Instead of a traditional ex post compensation scheme, the Swedish government intervened in 1996 in a way that reduced female wolverine poaching, which has probably contributed to the wolverine population recovery (Persson et al. 2015). In short, the program aims at rewarding the presence of reproducing wolverine females. For each wolverine female with cubs of the year that is verified (Fig. 3), reindeer herders in that district receive a performance payment that is supposed to match the value of livestock losses expected to occur over the year by wolverines, regardless of whether predation occurs or not. Thus, for the reindeer herder, every reindeer that is not killed by carnivores has twice the value of a reindeer that is predated, which creates an economic incentive to adopt husbandry practices that keep predation at a low level. Performance payment is only made when a female with cubs is verified by a trained ranger from the government according to standardized criteria in a national database. But before the verification can take place, the wolverine den has to be found. Reindeer herders cooperate very actively in wolverine monitoring together with the authorities and are often the ones who find dens or tracks that can then be verified and documented by the ranger.

The performance payment changes human behaviors by removing the moral hazard and reducing the motivation to poach by altering both behavioral and normative beliefs regarding wolverine conservation. The performance payment also feeds back into the ecological system by reducing poaching of females (Fig. 3) while poaching of males has not changed (Persson et al. 2015). By acknowledging the critical role of females to population viability, the program has been relatively successful in reducing poaching on the wolverine population. Because adult females produce female offspring who can disperse, the income-generating denning females propagate to potentially elevate profits or spread profits to neighboring villages. The Swedish performance payment program reduces wolverine-related costs to reindeer management districts and human-related costs to wolverines, and addresses several of the drawbacks of ex post compensation programs. However, it is important to note that Sami reindeer herding is enmeshed in a complex web of social–ecological interactions that vary dynamically across space and over time (e.g., climate change degrading forage conditions for reindeer, Moen 2008), thus the efficacy of the performance payment may decrease in the future if it no longer fits the social and biophysical contexts (Keskitalo et al. 2016).

## Future research using SES framework for carnivore poaching

Identifying and quantifying the strength of feedbacks related to carnivore poaching is challenging. Furthermore, multi-scale and multi-disciplinary data needed to elucidate social–ecological interactions and feedbacks are difficult to come by and to integrate. A range of techniques exists to collect data on various factors and processes affecting poaching opportunity (Gavin et al. 2010). For example, the randomize response technique utilizes probability theory to ascertain the likelihood that a certain proportion of survey respondents engage in illegal activities, such as poaching, while also protecting respondent confidentiality by ensuring that they are not associated with responses to sensitive questions (Nuno and St John 2015). Evaluating each of these techniques for data collection is beyond the scope of this paper. Instead, we briefly outline some examples of future research directions that can improve our understanding of feedbacks and inform interdiction of poaching in SESs.

Co-development of a site-specific poaching SES framework between researchers and local organizations can help identify and test key linkages in the SES that influence poaching (Steinmetz et al. 2006; Steinmetz and Srirattanaporn 2014), as well as the types of feedbacks (e.g., through addition or deletion of key actors) that emerge (Larrosa et al. 2016). Based on the context (see examples in Figs. 2 and 3), the SES framework can help develop specific research questions and hypotheses on how poaching opportunity changes with respect to changes in different attributes in the system. For example, how do different motivations to poach interact with carnivore population dynamics, and how do those interactions in turn feed back into human motivations? Dynamic models ideally can examine feedbacks among poaching rates, would-be poacher search efficiencies, guardian capabilities, and target animal behaviors, interacting with relative abundances of all actors. Furthermore, incorporating datasets from disparate disciplines, such as georeferenced human attitude data, into spatial models of poaching could improve our understanding of how landscape features and human psychology interact in space to influence poaching opportunity.

Computer modeling and scenario testing can be effective tools for designing, monitoring, and evaluating conservation interventions (Pressey et al. 2007; Bunnefeld et al. 2011). Understanding policy effects on poaching in SESs requires novel modeling strategies that integrate factors related to human motivations and animal vulnerability. For example, management strategy evaluation uses simulation models to test the future effects of alternative policies on species population dynamics under human exploitation (Bunnefeld et al. 2011). In addition, multi-agent-based models simulate the lives and behaviors of individuals and therefore can evaluate feedback effects among different policies and practices, human decision-making processes (e.g., micro-economic, social–psychological), and animal movements and distributions on poaching opportunity (Miller and Page 2007). Importantly, both of these modeling strategies can include, and have included, stakeholder participation in model development, helping to facilitate information sharing and collective learning. When integrated with insights from criminology, such modeling exercises could better assess the efficacy of a range of anti-poaching policies under changing and uncertain future conditions and improve decision-aid tools (e.g., decision trees).

## Conclusion

The growing complexity and global nature of poaching for the global commercial market threaten recovery efforts worldwide and are outpacing efforts to understand and address its effects. Understanding and addressing large carnivore poaching are especially challenging, because carnivore poaching is driven by a range of motivations influenced by various social–ecological dimensions spanning from individual to broader social and landscape levels. Emotions, cognitions, and livelihoods, among other attributes, interact to influence individual motivations to poach. Likewise, the behaviors, space use, and life-history traits, among other attributes, of individual animals interact to influence their vulnerability to poaching. Furthermore, the social, economic, and ecological conditions affect the behavior of individual actors—human and wildlife. These environmental conditions define the context in which behavior occurs, and shape it in ways that can be counterintuitive.

Based on this review, we developed a SES framework for carnivore poaching that ties together many of the factors across levels that influence poaching. The framework is useful in several ways. As with most major problems that lie at the intersection of social and environmental contexts, the framework suggests the high likelihood of feedbacks among systems of interactions between people and wildlife. The framework offers a common platform to help guide future research on poaching feedbacks, which has hitherto been lacking. Explicating these social–ecological interactions and feedbacks will likely help disentangle some of the complex features of carnivore poaching that has hindered effective global responses to the problem. The framework also provides entry points for site-specific studies through which more detailed measures and variables can be derived and analyzed. With continuing efforts to connect insights about poaching from disparate fields, we anticipate that the framework can be applied to other large mammal species, such as rhinoceros and elephant species, and can be the conceptual bases upon which a course for more effective and comprehensive poaching policy interventions is laid out.
